# Vegetation structure and greenness in Central Africa from Modis multi-temporal data

**DOI:** 10.1098/rstb.2012.0309

**Published:** 2013-09-05

**Authors:** Valéry Gond, Adeline Fayolle, Alexandre Pennec, Guillaume Cornu, Philippe Mayaux, Pierre Camberlin, Charles Doumenge, Nicolas Fauvet, Sylvie Gourlet-Fleury

**Affiliations:** 1 CIRAD, BSEF, 34098 Montpellier, France; 2 Gembloux Agro-Bio Tech, Université de Liège, 5030 Gembloux, Belgium; 3 SIRS, 59650 Villeneuve d'Ascq, France; 4 IES-JRC, European Commission, 2120 Ispra, Italy; 5 CRC, Biogéosciences, UMR 6282 CNRS/ Université de Bourgogne, 21000 Dijon, France

**Keywords:** remote sensing, tropical rainforest, Central Africa

## Abstract

African forests within the Congo Basin are generally mapped at a regional scale as broad-leaved evergreen forests, with the main distinction being between terra-firme and swamp forest types. At the same time, commercial forest inventories, as well as national maps, have highlighted a strong spatial heterogeneity of forest types. A detailed vegetation map generated using consistent methods is needed to inform decision makers about spatial forest organization and their relationships with environmental drivers in the context of global change. We propose a multi-temporal remotely sensed data approach to characterize vegetation types using vegetation index annual profiles. The classifications identified 22 vegetation types (six savannas, two swamp forests, 14 forest types) improving existing vegetation maps. Among forest types, we showed strong variations in stand structure and deciduousness, identifying (i) two blocks of dense evergreen forests located in the western part of the study area and in the central part on sandy soils; (ii) semi-deciduous forests are located in the Sangha River interval which has experienced past fragmentation and human activities. For all vegetation types enhanced vegetation index profiles were highly seasonal and strongly correlated to rainfall and to a lesser extent, to light regimes. These results are of importance to predict spatial variations of carbon stocks and fluxes, because evergreen/deciduous forests (i) have contrasted annual dynamics of photosynthetic activity and foliar water content and (ii) differ in community dynamics and ecosystem processes.

## 1. Introduction

Climate and land-use changes have modified the structure and productivity of ecosystems worldwide. In the next decades, African forests are predicted to experience profound climatic changes with increased temperature, alteration of rainfall patterns and possibly longer dry seasons [1–3]. There is thus an urgent need to have a better understanding of how current climatic conditions control vegetation structure and productivity, so as to predict the response to the ongoing climate change. Predicting responses requires first a good knowledge of the spatial distribution and characteristics of forest types, and second a better understanding of what drives the functioning of these forests.

In Central Africa, national vegetation maps (see Letouzey [4] for Cameroon, Boulvert [5] for the Central African Republic, Bégué [6] for the Republic of Congo) differ greatly between countries, in terms of detail, scale and floristic/functional terminology. These maps, as well as the large-scale forest inventories whose development have been led by timber concessions [7] nevertheless evidence strong spatial variations in species and trait distributions [8], forest composition [7] and forest structure such as above-ground biomass [9,10]. These patterns are in part associated with contrasted geological and soil features. A huge sandstone plateau crossing the border of CAR and the Republic of Congo has been shown to be a major landscape feature in the area, filtering species with a particular set of functional traits such as slow growth rates, high shade tolerance, evergreen leaves and high wood density [8]. Such characteristics are likely to interact with climate change to determine forest evolution in the future, and need to be mapped at the regional scale. Existing regional maps tend to gather the Central African forests within a ‘large broad-leaved evergreen forest’ category, distinguishing between terra-firme and swamp forest types [11,12], but such broad classes are not enough to help decision-making. A more detailed map evidencing forest types that may respond differentially to climate drivers remains to be produced.

Recent improvements in remote-sensing sensors, such as MODIS (moderate resolution imaging spectroradiometer), give access to the structure and greenness (photosynthetic activity), thus productivity, of tropical ecosystems [13–16]. Basal area and deciduousness are two key characteristics of tropical forests that need to be considered when identifying forest types with contrasting structure and greenness. The seasonality of photosynthetic activity can be remotely sensed at various scales and thus mapped over large areas [17]. Satellite time series across the year provided by MODIS instruments can help identify forests at a continental scale [18].

The climate drivers of forest greenness in Amazonia have been hotly debated [19,20]. It has been reported that forest greenness is maximum during the dry season when water availability is low but light availability is high [15,16]. However, the report by Saleska [21] on the green-up in Amazonia during the extreme drought during 2005 has recently been criticized [22]. In Africa, mean values of rainfall are lower than in Amazonia, and the rainfall regime is characterized by a double dry/wet period alternation on most of the Congo Basin [23,24], while a single alternation of a long dry period and a long wet period occurs in Amazonia. Light availability has been little documented [25] and both the patterns and drivers of African forests’ greenness remain to be clarified.

We thus had two aims in this study: (i) to identify spatial patterns of vegetation structure and greenness in Central Africa based on MODIS multi-temporal data, and validate it with forest inventory data and an existing national vegetation map and (ii) to evaluate the impact of current rainfall and light regimes on vegetation greenness. The results should provide decision-makers with a tool to better predict Central African forest resilience facing future climate changes.

## 2. Material and methods

### (a) Study area

**Figure 1.**
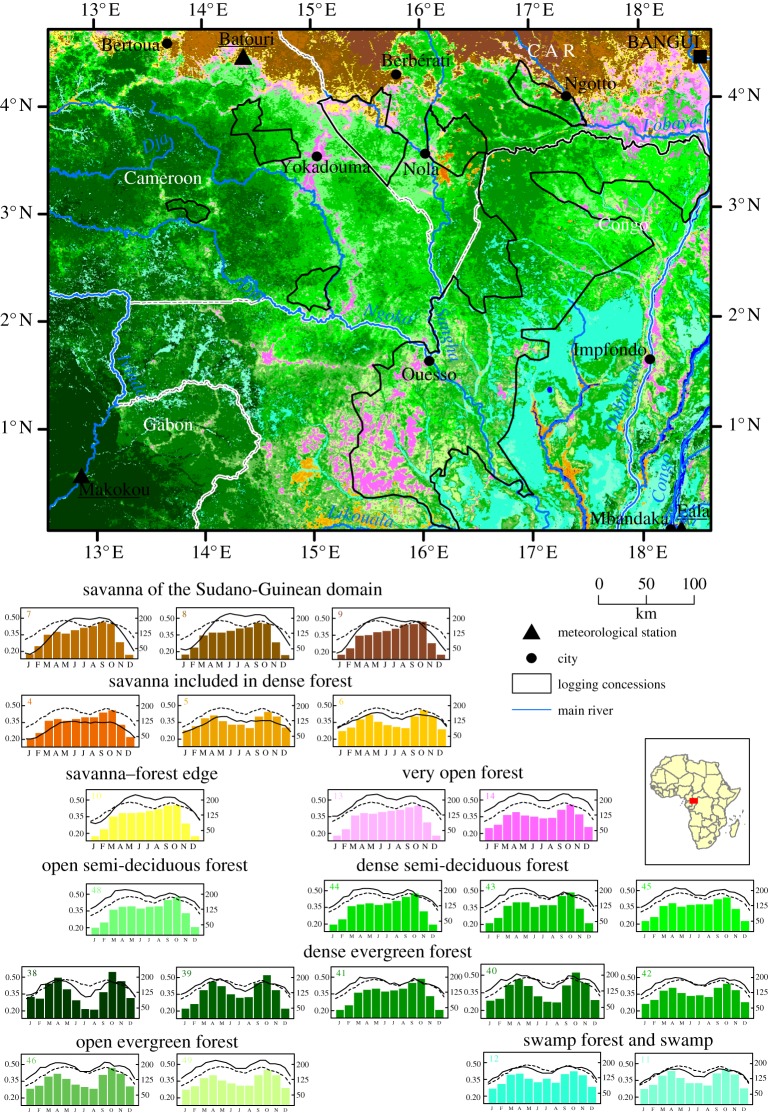
Vegetation map in the study area. The mean EVI profile is given for each class in the legend (solid line) as well as the mean profile over the study area (dashed line). Bars represent monthly rainfall.

The study area covers 30 million hectares (latitude 0°–5° N and longitude 13°–19° E) distributed over south-eastern Cameroon, southern CAR, north-eastern Gabon and northern Republic of Congo (figure 1). The climate is tropical humid across the study area, with a mean annual rainfall of 1400–1700 mm. The rainfall seasonality is driven by the inter-tropical convergence zone (ITCZ) that crosses the study area twice a year during equinoxes. Altitude ranges from 300 to 800 (m).a.s.l. The vegetation belongs to the Guineo-Congolian centre of endemism [26].

The density of the human population is very low in the study area (less than 10 inhabitants per km² except in the surroundings of Bangui; http://www.afripop.org/ and figure S1 in the electronic supplementary material). Selective logging [27] and some clearance for cultivation occurs.

### (b) Remote-sensing data

To quantify greenness, or photosynthetic activity, we used the enhanced vegetation index (EVI) data from the MODIS sensor. EVI was extracted from the ‘16-Day L3 Global 500 m product (MOD13A1 c5)’ from January 2000 to December 2009. EVI is directly related to photosynthetic activity [16,28] and compared with other vegetation indices such as NDVI that quickly saturates for high values of chlorophyll activity. EVI provides improved sensitivity for high biomass areas such as tropical forests [15,29].

To separate forest from non-forest vegetation types (mainly savannas and very open forests), we used the Surface Reflectance satellite data ‘8-Day L3 Global 500 m product (MOD09A1 c5)’ from the MODIS sensor to calculate the shortwave infrared water stress index (SIWSI) for the same period of time. SIWSI is related to leaf water content [30] and allows the discrimination of forest (with low canopy water content amplitude between dry and rainy seasons) from non-forest vegetation types (with high canopy water content amplitude between dry and rainy seasons). Both EVI 16-day and SIWSI 8-day composites are based on the minimum blue band reflectance, which reduces atmospheric biases.

We reconstructed a 10 year time-series mosaic and then performed a two-step classification approach of the newly built EVI and SIWSI datasets. During remote-sensing processing, even when composite images are used to reduce atmospheric and angular artefacts, pixels contaminated by clouds can persist and lead to strong misinterpretations [22]. To eliminate remnant clouds in the 10 year EVI dataset, we computed for each 16 day period the average value of the 10 satellite images available for each 16 day period (figure S2 in the electronic supplementary material). We thus obtained a mean EVI seasonal profile across a synthetic year. This process was not sufficient for the SIWSI dataset. We replaced the algorithm dedicated to EVI dataset by another one dedicated to SIWSI dataset. For each 8 day period and spectral band among the 10 year dataset, we retained the minimum pixel value that was the least likely to be affected by atmospheric artefacts. Temporal smoothing was then performed based on a simple linear interpolation designed to remove and replace contaminated pixels. We thus obtained a minimum SIWSI seasonal profile across a synthetic year.

To identify vegetation types with contrasted structure and greenness, we used a two-step classification approach of the EVI and SIWSI datasets both combining (i) an unsupervised ISODATA (iterative self-organizing data analysis technique) classification and (ii) visual interpretation of the results. The ISODATA classification is a K-mean algorithm which allows selecting clusters by splitting and merging the initial pixels datasets. The main advantage of this technique is the stabilization of the number of classes when the gravitational centre of the classes could not be split any more. We used an unsupervised classification because of the lack of training data for the whole area. This algorithm has already been successfully used for similar purpose in Madagascar [31]. Iterative work on two parameters (maximum number of iteration and maximum range of resulting classes) was done by empirically modifying their values to optimize the results of the classification, until it fully visually matched with Mayaux's [11] forested and non-forested patches delineation. This classification of the area of interest was performed by using Envi v. 4.3 software ([32]; Envi v. 4.3 software, Research Systems Inc.). The EVI dataset was used to identify forest classes, whereas the combined EVI–SIWSI datasets were used to improve the distinction among non-forested classes (including savannas, open forests and swamp forests).

The two initial parameters selected after our iterative work, were 20 iterations and 150–160 classes to free the positioning of the gravitational centre of classes. The classification on EVI data which best visually matches with Mayaux's map [11] identified 56 final classes corresponding to the stabilization of spectral and temporal variability of the data during the classification process. From this classification, we kept 11 forested classes (from numbers 38 to 49; figure 1) out of 56 classes in total, by comparing with Mayaux's map [11]. The 45 other classes were merged and used as a non-forest mask to extract the EVI–SIWSI dataset. The second classification, which was run on this non-forest mask, using EVI–SIWSI dataset stabilized at 14 final non-forested classes with initial parameters of 10 iterations and 25–30 classes. From this classification, we merged three of them in a water class (from class 1 to class 3) due to high spectral variability of this feature. To make a comparison with other spatial information (vegetation map, inventory plots), we projected the classification into a Universal Transverse Mercator projection (zone 31, ellipsoid WGS84).

### (c) Forest inventories and vegetation map

To validate the remote-sensing analyses, we used two sources of information: commercial forest inventories and the vegetation map of Cameroon [4].

Commercial forest inventories were conducted over the 2000–2007 period within 19 logging concessions located in Cameroon, CAR and Congo (see the electronic supplementary material, figure S3). All companies used a similar systematic sampling design, where all trees with a diameter of 30 cm or more at breast height (dbh) were recorded in parallel transects 2–3 km apart and divided into 0.5 ha consecutive plots [7–9]. A total of 38 020 plots (6 million hectares) individually geo-positioned were available for this study. In each 0.5 ha plot, trees with diameters up to 140 cm were assigned to 10 cm dbh classes and larger trees were grouped in the more than or equal to 150 cm dbh class. We first computed the total plot basal area, using the mean diameter for each diameter class. Vernacular names used in field inventories were converted to genus level scientific names. A total of 339 genera were identified in the 38 020 plots. In this study, we restricted the analysis to the 197 genera that were observed with a frequency of over 1%. Information on leaf phenology deciduous versus evergreen) was collected for from floras for 402 African tree species. We assigned a leaf phenology at the genus level (dominant leaf phenology across congener species) for 171 genera among which 116 were considered evergreen and 55 deciduous. Deciduousness was calculated at the plot level as the proportion of stems with a deciduous phenology. When there was no clear evidence at the genus level, the genus was ignored in the trait calculation. In this study, we only consider the 37 898 plots for which leaf phenology was available for more than 70% of the stems and 90% of plot basal area.

Because we had access to limited inventory data in the Cameroonian area, we used the detailed vegetation map of Letouzey as a field validation dataset.

### (d) Climatic data

We used the products derived from the FEWSNET rainfall estimation (RFE) decadal imagery (http://earlywarning.usgs.gov/fews/africa/index.php [33]) at 8 km spatial resolution. Monthly rainfall estimation was computed and then a monthly average from 2000 to 2008 was calculated. A comparison with lower-resolution rainfall datasets based on rain gauge data revealed that the RFE averages were satisfactorily reproducing the local rainfall regimes (not shown).

We obtained ground measurements of light intensity from three meteorological stations (figure 1): Eala in northwest Congo (0°03′ N and 18°17′ E; 320 (m).a.s.l., from January 1957 to December 1959); Makokou in northeast Gabon (0°33′ N and 12°51′ E; 500 (m).a.s.l., from January 1951 to December 1975) and Batouri in southeast Cameroon (4°26′ N and 14°22′ E; 630 (m).a.s.l., from January 1983 to June 1985). Monthly averages were then computed. These data are not synchronous with the MODIS dataset.

### (e) Data analysis and validation

**Figure 2.**
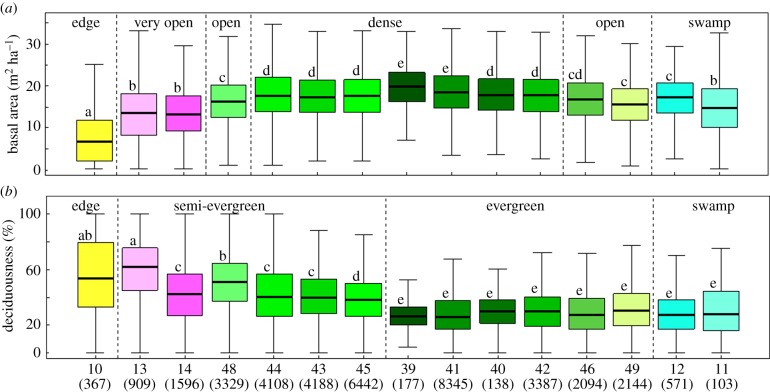
Differences in (*a*) basal area and (*b*) deciduousness among forest classes. Different lowercase letters (*p* < 0.001) indicate significant differences in the pairwise Wilcoxon test. The number of 0.5 ha plots is indicated in brackets below the class number. Colours of symbols correspond to figure 1.

To validate the map, we examined whether the classes evidenced by the classification procedure differed in terms of structure and greenness. For this, we assigned the remotely sensed vegetation classes to each available inventory plot. We then tested for differences in plot basal area and degree of deciduousness among classes with pairwise Wilcoxon tests and Bonferroni's adjustment for multiple comparisons. We restricted this analysis to the classes characterizing forests, represented by more than 100 0.5 ha plots (figure 2). In Cameroon, where we did not have access to enough inventory data, we examined the consistency of the classes with the vegetation types evidenced by Letouzey [4].

To obtain EVI and rainfall seasonal profiles, we first averaged EVI values and rainfall for all pixels per vegetation class. Indeed, rather than monitoring EVI dynamics of each pixel we extracted this information from groups of related pixels. The process is based on a spatial average. All statistical analyses were conducted within the R environment [34].

## 3. Results

With the two-step classification of EVI and SIWSI datasets, we identified and mapped 22 vegetation types (we took out the three water classes merged into one). Within these 22 vegetation types, six savannas, three open forests, 11 dense forests and two swamp forests were delineated and labelled thanks to the interpretation of the EVI signal shape (figure 1, table 1) and visual interpretation [11]. We identified strong variations in stand structure and deciduousness across these types (figure 2). Among them, we recognized (i) six classes of savanna (MODIS classes 4–9). We distinguished the savannas of the Sudano-Guinean domain from included savannas that sparsely occurred in the forest matrix; (ii) one class of savanna—forest edge (MODIS class 10), which exhibited the lowest value of basal area (7.5 m² ha^−1^) and one of the two highest values of deciduousness; (iii) two classes of very open forests (classes 13 and 14), which exhibited the second lowest values of basal area (13.5 m² ha^−1^) and were both semi-deciduous with 57% and 41% of deciduous stems, respectively. These types corresponded to degraded forests along roads and close to main cities and to *Marantaceae* forests that cover a huge area in the north of the Republic of Congo, south of Ouesso; (iv) eleven classes of forests, comprising open and dense forests: one open (16.5 m² ha^−1^) semi-deciduous (48%) forest class (MODIS class 48); a group of dense semi-deciduous forests (approx. 40% deciduousness, MODIS classes 43, 44 and 45) and a group of dense evergreen forests (less than 30% deciduousness, MODIS classes 39, 40, 41, 42); two classes of open evergreen forests (MODIS classes 46 and 49), exhibiting low deciduousness similar to that of a dense evergreen forest but with lower basal area (17.1 and 15.7 m² ha^−1^, respectively). Finally, one dense evergreen forest (MODIS class 38) was not documented by field inventories; (v) two classes of swamp forests (MODIS classes 11 and 12), which had both low deciduousness but differed in basal area. They are mainly located in the Congo Basin and along main rivers elsewhere. Validation of the forest types was performed using field inventories (figure 2) and Letouzey's map in Cameroon (see the electronic supplementary material, table S1).

**Table 1.** Description of MODIS classes. Mean and s.d. of EVI, percentage of good quality observation (pixel reliability obtained from NASA documentation) and sum and s.d. of rainfall are given for each class, so as the *R*² indicated the fraction of variation in EVI explained by rainfall.

MODIS classes: number and description		EVI			rainfall (mm)		*R*²
		mean	s.d.	obs. %	sum	s.d.	
3	open water: rivers, lakes	0.25	0.032	0.95	1454	25	0.34
7	savanna of the Sudano-Guineanian domain (north)	0.38	0.123	0.91	1384	55	0.83
8	savanna of the Sudano-Guineanian domain (south)	0.42	0.120	0.91	1368	55	0.80
9	savanna of the Sudano-Guinean domain between Berberati and Mbaiki	0.40	0.109	0.94	1376	57	0.80
4	savanna included in dense forests	0.31	0.058	0.86	1439	44	0.83
5	savanna included in dense forests, along main rivers and Marantaceae forests (south Ouesso)	0.34	0.042	0.88	1469	29	0.26
6	savanna included in dense forests, along main rivers and Marantaceae forests (north Congo)	0.39	0.038	0.87	1443	33	0.43
10	savanna-forest edge mixed with agriculture	0.45	0.089	0.90	1383	55	0.82
13	very open forests mixed with agriculture	0.48	0.066	0.92	1388	52	0.85
14	very open forests located along roads and Maranthaceae forests (south Ouesso, north Congo)	0.51	0.048	0.93	1418	34	0.71
48	open forests closed to main roads and cities	0.48	0.050	0.91	1430	49	0.69
44	dense semi-deciduous forests mostly located in CAR	0.46	0.054	0.92	1410	50	0.72
43	dense semi-deciduous forests in the Sangha River Interval	0.46	0.047	0.88	1447	47	0.63
45	dense semi-deciduous forests mostly located in the Oubangui Basin	0.47	0.051	0.92	1399	38	0.84
38	dense evergreen forests in Gabon	0.41	0.054	0.59	1531	59	0.59
39	dense evergreen forests in Cameroon	0.43	0.043	0.71	1506	53	0.72
41	dense evergreen forests with a disjunctive spatial distribution:	0.44	0.045	0.89	1444	48	0.70
	— south CAR and north Congo						
	— western limit of class 40 in Cameroon						
40	dense evergreen forests located at the western limit of 38 and 39	0.44	0.046	0.73	1536	47	0.57
42	dense evergreen forests at the edge of swamp forest in north Congo	0.45	0.044	0.91	1449	31	0.71
46	open evergreen forests mixed with swamp forest in north Congo	0.47	0.045	0.88	1475	35	0.49
49	open evergreen forests located in north Congo closed to rivers and Marantaceae forests	0.49	0.046	0.92	1435	31	0.58
12	swamp forests located in the Congo Basin	0.43	0.040	0.92	1445	28	0.73
11	swamp located at the valley bottom in the Congo Basin and along rivers in Cameroon and Gabon	0.41	0.039	0.84	1477	34	0.68

For all vegetation classes, mean EVI profiles were highly seasonal and correlated to rainfall seasonality (figure 1, table 1). Savannas of the Sudano-Guinean domain showed the greatest seasonal variations with the highest standard deviation of EVI values. Savannas included in dense forest showed the lowest mean EVI values, whereas the open degraded forests (class 14) had the highest mean EVI values. The EVI profiles of all forest classes showed two peaks corresponding to the two rainy seasons of March–May (short rainy season) and September–November (long rainy season), alternating with two periods of lower EVI values, in December–February (long dry season) and in June–August (short dry season).

The three stations showed similar temporal profiles between EVI and light intensity with little time lag between respective maximum and minimum values (see the electronic supplementary material, figure S4).

## 4. Discussion

In this study, we aimed to identify spatial patterns of vegetation structure and greenness in Central Africa. Specifically, we identified a wide spectrum of tropical vegetation types (savannas, forest and swamps) and strong spatial variations in stand structure and deciduousness across forest types. All vegetation types were described with more than 90% of good quality acquisition, except for the westernmost classes that were affected by clouds during calculation (table 1). Future studies focusing on the western part of Central Africa should thus pay attention to this effect and take into account the quality assessment of the MODIS data during similar processing.

Two main blocks of dense evergreen forests, exhibiting a low level of photosynthetic activity, were detected in the area. In southern CAR and in the north of the Republic of Congo, forest growing on a sandstone plateau showed much lower deciduousness than climatically expected. These results are in strong agreement with the results of Bohlman [35], who reported that deciduousness decreases with increasing annual rainfall, but also that geology can alter this relationship [8]. In the western and wettest part of the study area, the dominance of evergreen forests is probably driven by climate rather than particular geological substrate.

Semi-deciduous forests associated with a high level of photosynthetic activity dominated large parts of the study area. The location of these semi-deciduous forests matches with the Sangha River Interval, a 400 km wide area (14–18° E) characterized by a low endemism, which has experienced major vegetation changes in the past [36]. Forest fragmentation has occurred as recently as during the Holocene (approx. 2500 BP) followed by the expansion of pioneer and secondary species [37,38]. Furthermore, the opening of such a ‘corridor’ emerged from recent vegetation modelling of different scenarios of climate changes [2], and corresponds to the very open forests where human activities are important along roads [7].

Our findings fit well with national vegetation maps in the study area. In CAR, the location of evergreen forests typical of the sandstone plateau is correctly mapped [5,7,9]. The open *Marantaceae* forest identified by Bégué in northern Congo [6] is also well located. The spatial arrangement of forest types is in accordance with Letouzey [4] in Cameroon. Our results helped translate previous heterogeneous information into a homogeneous map evidencing forest types based on their structure and greenness, i.e. suitable for studying their link with environmental drivers.

Moreover, we showed that, in the studied area, forest greenness was highly seasonal and strongly correlated with rainfall and to a lesser extent to light seasonality. The EVI seasonal profiles of all vegetation types identified (including swamp forests) were nearly the mirror image of the seasonal patterns of rainfall and light availability. These findings of strong rainfall and light control on EVI profiles in Central Africa contrast with that of Myneni [15], who showed a seasonal increase in the leaf area of the Amazon forest during the dry season when irradiance is maximal and rainfall is minimal. In Central Africa, the movement of the ITCZ generates two rainy and sunny seasons and two contrasting dry seasons. The unexpected light intensity during the rainy seasons, results from the fact that the sky is clear in the morning. The induced surface warming later in the day promotes atmospheric instability and convection. Convective clouds develop during the afternoon, leading to thunderstorms that do not occur until late afternoon or at night. The association between high rainfall, high light intensity and high EVI level suggested that these periods were optimal for photosynthetic activity. Seasonal changes in light intensity have also been shown to strongly drive tree phenology in Central and Southern America [39,40]. However, the role of light availability on tree phenology and forest functioning in Africa remains to be examined, since light intensity data were only available for three sites and the date ranges tend to be much earlier than for the MODIS data. Moreover, swamp forests that are not limited by water availability react such as terra-firme forest, suggesting their dependence to light intensity.

In Central Africa, where climatic variability is low [23,41,42] and annual rainfall is spatially homogeneous, any modification in dry season length and intensity could have dramatic consequences on vegetation structure and greenness [43]. Some slight climatic differences might be more important than previously thought. In the study area, differences in vegetation phenology and traits have been evidenced and these can be linked not only to differences in soil properties, but also to slight differences in climate variables which may have been overlooked. The distribution of semi-deciduous and disturbed vegetation is in line with the Sangha River Interval, a region that has probably experienced more impact of past climate changes than elsewhere in the study area. In fact, the Sangha sub-river basin of the Congo River has regularly received less precipitation between 1950 and 1980 than the Oubangui and Central Congo sub-basin bordering it [24]. Although new and more detailed data are necessary to confirm this, we can tentatively conclude the relative fragility of this area in the face of climate changes, notably precipitation decrease and/or dry season increase.

Tropical forests do not necessarily function similarly across the globe, and it is crucial to identify the differences in the effects of the current climate on the functioning of located and characterized tropical forests in order to forecast responses to climatic changes. We showed that the same drivers (water and light) determine forest greenness in Africa and Amazonia but the relative importance of water may be greater in Africa [15]. Central Africa, on a seasonal basis, combines high EVI with high rainfall, and high light intensity, whereas Amazonia combines high EVI with low rainfall and high light intensity [16,17]. Central Africa has lower annual rainfall than Amazonia, and this may influence the specificity of its forests [44]. Dense evergreen and semi-deciduous forests are driven by rainfall and light regimes, making them vulnerable to changes in rainfall and light amounts and dry season length. In the context of climate change and increasing anthropogenic pressure [45], these specificities have to be borne in mind with regard to future management and conservation policies.

## Data accessibility

We wish to thank the timber companies Alpicam, BPL, Danzer, DLH, IFB, Likouala Timber, Rougier, SEFCA, SCAD, SCAF and Vicwood for authorizing access to the inventory data.
